# Incidental Intercostal Intramuscular Myxoma With Fibrous Dysplasia in a Patient With Mazabraud’s and McCune Albright Syndromes

**DOI:** 10.7759/cureus.22869

**Published:** 2022-03-05

**Authors:** Ali S Tejani, Vishal Kukkar

**Affiliations:** 1 Radiology, University of Texas (UT) Southwestern Medical Center, Dallas, USA; 2 Radiology, University of Texas (UT) Southwestern Medical School, Dallas, USA

**Keywords:** cardiothoracic radiology, mccune albright syndrome, mazabraud’s syndrome, fibrous dysplasia, intramuscular myxoma

## Abstract

Intramuscular myxoma is a rare entity that may present as single or multiple lesions in patients with Mazabraud’s syndrome and is characterized by intramuscular myxomas with fibrous dysplasia. Though intramuscular myxomas occur in large muscle groups, they can very rarely occur in the chest wall. We present the case of a 41-year-old woman with an incidentally discovered intercostal mass on magnetic resonance cholangiopancreatography (MRCP). Repeat MRI demonstrated a lobulated, T2-hyperintense intercostal lesion and demonstrated adjacent fibrous dysplasia of the ribs, consistent with the patient’s history of Mazabraud's and McCune Albright syndromes. Histopathological exam following surgical resection confirmed a diagnosis of intramuscular myxoma without the presence of sarcomatous changes. Though small, slow-growing intramuscular myxomas may be observed with conservative management in the absence of significant symptoms, surgical resection is warranted to prevent complications such as osseous erosion or nerve impingement.

## Introduction

Myxomas are rare, benign mesenchymal tumors that can occur in various anatomic locations either independently or as a constellation of pathologic syndromes. Though the most frequent site for myxomas is within the myocardium, extracardiac myxomas can occur most commonly in musculature, primarily in large muscle groups [1]. Very rarely, these soft tissue tumors can occur in other locations such as the chest wall. Intramuscular myxomas are mesenchymal in origin and present as indolent, slowly enlarging masses with a predilection for female and middle-aged patients [1]. Single or multiple intramuscular myxomas can be seen in Mazabraud’s or McCune Albright syndromes, characterized by accompanying single or multiple bone fibrous dysplasias. Intramuscular myxomas in the setting of Mazabraud’s syndrome often occur within close proximity of associated fibrous dysplasia, as opposed to large muscle groups in the extremities when seen in isolation [2-3]. In this report, we describe the case of a 41-year-old female patient with a history of Mazabraud’s and McCune Albright syndromes who presented with an incidentally discovered intercostal mass on magnetic resonance cholangiopancreatography (MRCP) with adjacent fibrous dysplasia of the ribs, which was found to be histologically consistent with an intramuscular myxoma.

## Case presentation

A 41-year-old woman with a history of Mazabraud’s and McCune Albright syndromes and known polyostotic fibrous dysplasia, multiple endocrinopathies (Cushing syndrome, status post adrenalectomy, and hypothyroidism), and multiple prior symptomatic myxomas, presented for the evaluation of an incidentally discovered right intercostal mass between the seventh and eighth ribs on MRCP performed for follow-up imaging of known pancreatic cysts and surveillance for intraductal papillary mucinous neoplasm. She notes that she has not had any recent pain associated with the right chest wall, and she has not noticed any enlarging, palpable mass. Relevant medical and surgical history includes multiple prior gluteal myxomas with surgical resection for worsening pain, resulting in symptomatic relief. Additionally, the patient reported ongoing evaluation for a slowly enlarging right neck myxoma causing right arm pain. Physical exam revealed a small, palpable right neck mass but did not reveal any palpable mass or tenderness along the region of interest at the chest wall.

**Figure 1:**
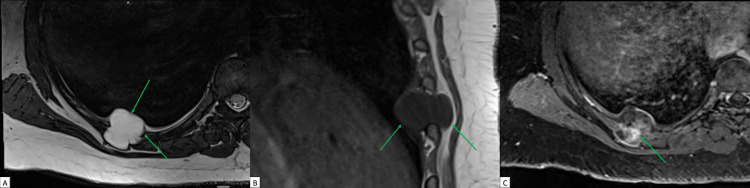
A 41-year-old, asymptomatic woman with Mazabraud’s and McCune Albright syndromes with incidental intercostal intramuscular myxoma (A) Axial T2-weighted MR image of the chest shows high signal intensity within a lobulated lesion in the 7th-8th intercostal space (arrows). (B) Sagittal T1-weighted MR image of the chest shows a homogenously hypointense lesion (arrow). (C) Axial contrast-enhanced T1-weighted image of the chest shows heterogeneous, patchy, delayed enhancement within the lesion (arrow).

**Figure 2:**
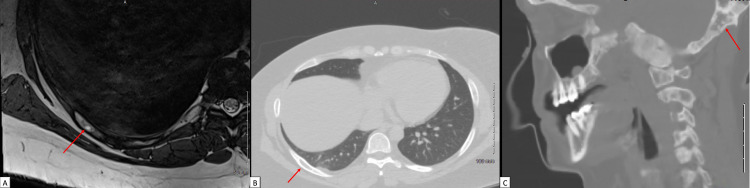
A 41-year-old, asymptomatic woman with Mazabraud’s and McCune Albright syndromes with an incidental intercostal intramuscular myxoma. Cross-sectional imaging demonstrating polyostotic fibrous dysplasia (A) Axial T2-weighted MR image of the chest shows hyperintensity and expansion (arrow) within the eighth posterior right rib. (B) The follow-up axial image from CT of the chest shows a well-defined expansile, cystic lesion (arrow) within the eighth posterior right rib. (C) The sagittal image from CT of the neck shows multifocal sclerotic and cystic changes notably involving the occipital bone (arrow), consistent with the patient’s known history of polyostotic fibrous dysplasia.

A review of the MRCP from one month prior demonstrated stability of her multiple cystic pancreatic lesions measuring up to 8 mm, without suspicious features, and revealed a significantly T2-hyperintense lobulated lesion measuring 3.1 x 2.7 x 2.5 cm in the posterior seventh to eighth intercostal space. Repeat MRI of the chest at the time of this visit confirmed this finding (Figure 1), as well as an enhancing fusiform enlargement of the lateral eighth rib correlating with expansile rib lesions on CT chest, consistent with the patient’s known polyostotic fibrous dysplasia (Figure 2). Core biopsy of the mass was performed, rendering a diagnosis of intramuscular myxoma with CD34 positive and S100 negative cells on immunohistologic stains.

**Figure 3:**
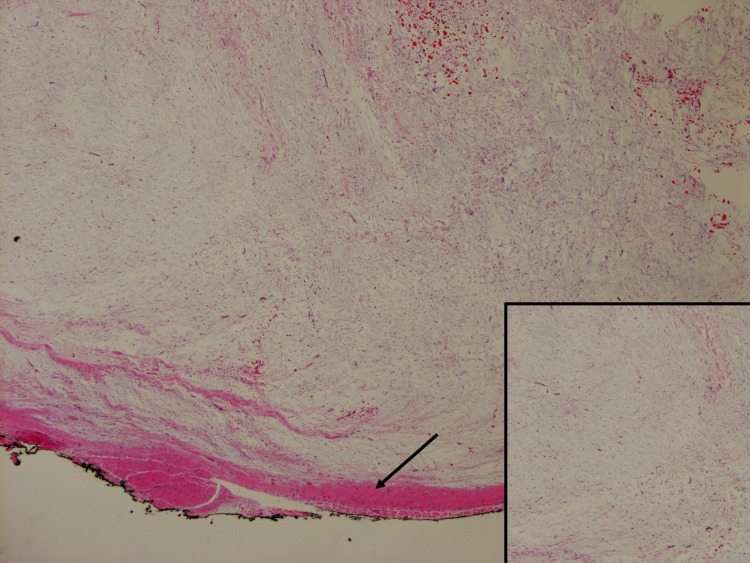
A 41-year-old, asymptomatic woman with Mazabraud’s and McCune Albright syndromes with an incidental intercostal intramuscular myxoma. Low-power view Low-power histological specimen following surgical resection demonstrating intramuscular myxoma. The lesion is well-circumscribed, hypocellular, hypovascular, and diffusely myxoid (inset image). Focal skeletal muscle is present at the periphery of the lesion (arrow).

**Figure 4:**
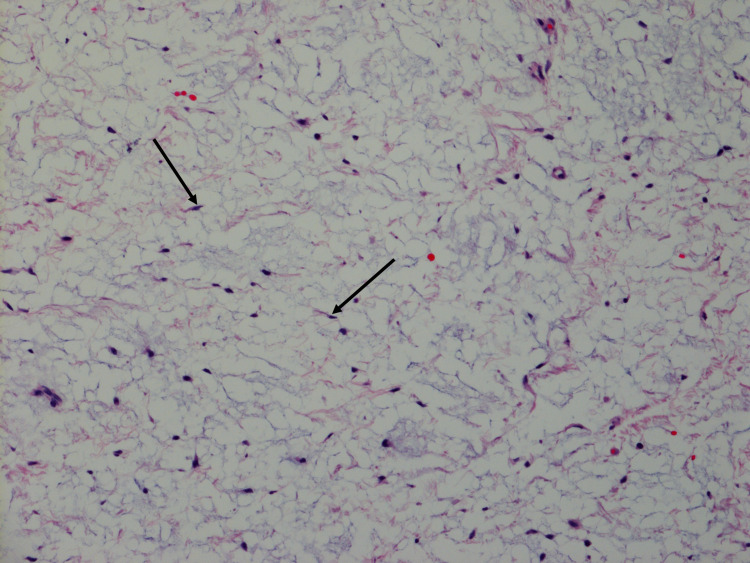
A 41-year-old, asymptomatic woman with Mazabraud’s and McCune Albright syndromes with an incidental intercostal intramuscular myxoma. High-power view High-power view of the histological specimen following surgical resection demonstrating intramuscular myxoma. Notable features include scattered small, bland, spindled cells (arrows) present in the abundant myxoid stroma.

Given the size of the tumor, from potential intercostal nerve impingement, and the patient’s history of multiple symptomatic past myxomas, the patient and her surgical team agreed upon surgical resection of the mass. During the procedure, a mass protruding from the intercostal muscle to the extrathoracic soft tissue was identified. Resection involved enucleation of the mass after incision of the intercostal muscle and adjacent parietal pleura. Macroscopic evaluation of the surgically resected specimen revealed an ovoid, gray-tan nodule with a gelatinous, hemorrhagic cut-surface measuring 4.2 x 2.5 x 2.0 cm. Histologic examination of the surgical lesion showed diffusely myxoid, hypocellular content within the nodule (Figure 3). High-power microscopic evaluation showed scattered spindle cells within the myxoid stroma (Figure 4).

The patient was admitted for pain management and discharged on postoperative Day 1 following her resection with a right-sided Jackson Pratt (JP) drain terminating in her pleural cavity. Her postoperative course was complicated by right-sided empyema secondary to *Serratia marcescens*, presenting as sepsis, requiring placement of three additional right-sided chest tubes and intravenous (IV) antibiotics. Treatment with antibiotics (IV vancomycin/piperacillin-tazobactam narrowed to IV cefepime followed by PO ciprofloxacin for a total of four weeks) improved her clinical course with a resolution of her symptoms. Her chest tube output gradually declined, and all chest tubes were removed without complication.

## Discussion

Intramuscular myxomas are a sub-type of soft tissue myxomas representing rare, benign tumors of mesenchymal origin with a predilection for middle-aged (i.e., ages 40-70) and female patients, as seen in the current case [1]. Intramuscular myxomas tend to present as indolent, slow-growing lesions in large muscle groups, particularly in the extremities, most commonly in the thigh, shoulder, buttocks, and upper extremity, rarely occurring in other locations such as the chest wall [4]. While lesions may present as isolated findings, multiple intramuscular myxomas can occur as a constellation of findings in Mazabraud’s or McCune Albright syndrome, associated with single (monostotic) and multiple (polyostotic) fibrous dysplasia or multiple endocrinopathies, in the latter. Though intramuscular myxomas have been described in Mazabraud’s syndrome previously, this case is the first to describe an intercostal intramuscular myxoma in a patient with both Mazabraud’s and McCune Albright syndromes [5]. As demonstrated in this case, tumors tend to occur near regions of fibrous dysplasia in the setting of these syndromes.

Typical imaging findings of intramuscular myxomas include homogenous, hypodense lesions with well-defined margins and soft-tissue attenuation on CT images [6]. Correlating MRI findings include hypointensity on T1-weighted images, hyperintensity on T2-weighted images, and varying patterns on contrast-enhanced phases, including heterogeneous or patchy and peripheral enhancement [1]. In patients with accompanying fibrous dysplasia, affected bones may show ground-glass, sclerotic, or cystic expansile and well-demarcated lesions [3]. MRI findings include varying patterns of heterogeneous signal on T1- and T2-weighted images, including heterogeneous contrast-enhancement [7]. MRI findings are less specific for fibrous dysplasia, and further evaluation for suspicious bony lesions with CT images can help narrow the differential with the added benefit of determining the extent of pathology for pre-surgical planning.

Histological diagnosis of intramuscular myxoma requires a hypocellular, hypovascular lesion with an abundant myxoid component [8-9]. Cells within the lesion are cytologically bland stellate or spindle cells without any evidence of atypia, mitoses, or pleomorphism. Immunohistological tests are positive for vimentin, CD34 (50% of cases), and smooth muscle actin (10% of cases) while negative for desmin and S100 [10]. Differentiation from more aggressive lesions, such as myxofibrosarcomas or angiomyxomas, relies on the absence of high cellularity, mitotic figures, and vessels within the lesion.

Small, slow-growing intramuscular myxomas may be observed with conservative management in the absence of significant symptoms [6]. However, surgical resection is warranted for worsening pain, osseous erosion, or neurological deficits owing to the mass effect from enlarging tumors. Wide local excision with tumor-free margins has been described as sufficient to ensure low rates of recurrence [11]. Associated risk factors for recurrence include increased cellularity on a histopathological exam [2].

Similarly, surgical intervention is warranted for fibrous dysplasia in the context of worsening mass effect from growing lesions, or from rare malignant transformation to osteogenic sarcoma, as noted in case reports of patients with Mazabraud’s syndrome [12-13].

## Conclusions

Intramuscular myxomas are rare lesions associated with Mazabraud’s and McCune Alright syndromes. Though these lesions tend to occur in large muscle groups, they may rarely present in the chest wall. When associated with these syndromes, intramuscular myxomas tend to occur near regions of fibrous dysplasia. Small, slow-growing intramuscular myxomas may be treated with conservative management; however, surgical intervention is warranted to prevent complications such as adjacent osseous erosion or nerve impingement from tumor growth.
